# Specific Features of Ethnic Identity in the Regions with Varying Degrees of Ethnic Diversity

**DOI:** 10.3390/bs10010013

**Published:** 2019-12-25

**Authors:** Olga Zotova, Lyudmila Tarasova, Olga Solodukhina, Natal’ya Belousova

**Affiliations:** Department of Social Psychology, Liberal Arts University (LAU)–University for Humanities, Surikova St 24a, Yekaterinburg 620144, Russia; tarasovagu@mail.ru (L.T.); avsuson@gmail.com (O.S.); nata.isk@mail.ru (N.B.)

**Keywords:** ethnic identity, ethnic diversity, poly-ethnicity, tolerance

## Abstract

Ethnic diversity describes the plurality of ethnicities within a group of people coexisting in one territory. The permanent presence of other cultures’ representatives can trigger a sense of jeopardy; a feeling that the prevailing way of life, its norms, and its values are challenged by strangers, which results in hostility to ethnic minorities living in the same territory. In this context, the study aimed at investigating specific features of the individual’s ethnic identity determined by the degree of the ethnic diversity of their living environment is of relevance. In order to define regions for the study, the comparative analysis of the ethnic diversity of Russian regions was conducted. Two regions for the study were defined: the Sverdlovsk region as a territory with average ethnic diversity and the Republic of Bashkortostan as a highly diverse region in terms of ethnicity. The respondents from less ethno-diverse areas exhibit global self-identification, the awareness of being a part of the world, and territorial identity. Differences in the degree of sustainability and the intensity of ethnic self-identification of the subjects from regions with varying degrees of ethnic diversity were revealed. Significant distinctions in the meaning of ethnicity for the compared groups of the respondents were found.

## 1. Introduction

We are living in a world of a growing medley, not only in terms of measurable objects, forms, and peculiarities, but also from the perspective of social arrangement, imagination, thoughts, and constructions of reality. The complexity has become an inevitable paradigm for effective research focused on the interrelated socio-economic, biophysical, and political systems in time and space [1,2,3].

A great number of modern theories and conceptions explain the pros and cons of this diversity [4,5]. The theory of sociological systems [6], for example, implies that the strengthening of the differentiation in social systems increases their ability to adjust to future challenges, the diversity of social structures and organizations leads to an overall economic progress [7]. On the other hand, the increasing social assortment requires additional social mechanisms of control and management, allocation of resources, and conflict settlement.

Objections and the dispute over what is good and right are inevitable in the contemporary academic community. Certainly, one can consider ethnic groups to be “imagined communities” while a nation is a product of peoples’ imagination and creativity [8]. However, despite the complexity and ambiguity of the definition of ethnic groups, research on this subject in terms of interrelations and dependencies on different socio-psychological phenomena is relevant and will remain so for decades to come.

Even by knowing that belonging to a particular group is temporary, a person can include this element of self-knowledge in their actual identity more or less likely. The identity build-up based on a person’s identification with those groups the membership in which depends on one’s own will and desire leaves more flexibility for self-identification and, to a greater extent, makes the person their author.

The ethnic diversity describes the plurality of ethnicities within a group of people coexisting in one territory. Ethnic groups can live together in a society or form cultural enclaves or “diasporas” within the territory of a receiving country. Ethnic diversity is often viewed as detrimental to social harmony and political stability. Governments in many countries have expressed concerns over ethnic diversity repeating their calls for national unity, assimilation of national minorities’ associations, and a reduced ethnic identity. Even gentle political actions are based on the purpose of sameness rather than a plurality of ethnic community, which is sometimes seen as backwards.

Intensive cultural mobility and ethnicity mix make the problem of preserving ethnic identity even more acute [9] (p. 825). In many multinational societies, social and political differences and discrepancies are marked across cultural (ethnic, racial, or religious) borders. People use ethnic belonging as one of the most understandable and available forms of group solidarity and as an instrument for achieving various goals. That is why ethnicity acts as a source of unrest in the world where power, prosperity, and rank among and within countries are allocated illegally and unequally.

How the host population perceives other nationals also depends on the cultural specificity of interethnic interaction. A permanent presence of other cultures’ representatives can trigger a sense of jeopardy; a feeling that the prevailing way of life, its norms and values are challenged by strangers [10,11], which results in hostility to ethnic minorities living in the same territory. In situations of conflict and confrontation, the choice of the migrants’ majority is to unite against a common threat from native citizens resulting in social segregation on grounds of ethnicity and risk of interethnic clashes [12] (p. 619).

The challenges of globalization revealed the problem of interethnic interaction as a key factor of social stability and security. Under current changes, ethnic identity is not static, but a dynamic formation that does not remain unchanged throughout the person’s life span. Social interactions, conflicts in polyethnic environments can often result in rethinking the role of a person’s ethnicity and the transformation of his ethnic identity.

Positive ethnic identity provides individuals with a sense of belonging, purpose, social support, and increased self-esteem [13]. This process can occur through attachment to one’s ethnic group in which beliefs and values are shared and reinforced by the group members [14]. Ethnic identity encourages sustainability, shapes pro-social styles of overcoming difficulties, and serves as a defense mechanism [15,16,17,18,19,20,21]. D.J. Brown, R.T. Cober, L.M. Keeping and P.E. Levy showed that from the perspective of ethnic diversity within an organization, racial tolerance is associated with such personal traits as openness to experience and self-esteem [22].

In living areas, ethnic boundaries are blurred due to social interactions with local people, which gradually results in accepting a host country’s culture. Ethnic minority in a region with a high density of another ethnos will have fewer stimuli for preserving their belonging, which, in turn, can enhance the process of the cultural integration.

Earlier empirical data about the linkage between ethnic segregation and the formation of ethnic identity have yielded meager and mixed results. According to A. Bisin and his colleagues, the identity of ethnic minorities can be more intense in the mixed regions rather than in the segregated regions [23]. By contrast, H. Battu and Y. Zenou provide evidence that life in an ethnic enclave is associated with a very low level of identification and quite a strong affiliation with a particular ethnic group [24]. A.M. Danzer and F. Yaman state that in the regions with low ethnic diversity, the intensity of social interaction between ethnic minorities and the local population decreases [25].

Clearly, ethnicity shares many links with time, context, and a multitude of circumstances. People often appeal to it when they feel uncomfortable in the world around them, striving to find a way out of uncertainty by joining a community with distinct boundaries and the divide between ‘us’ and ‘them’.

Thus, social interactions, for instance, conflicts within a polyethnic environment and can cause us to rethink the role of ethnic belonging and ethnic identity transformation. These problems are especially acute for representatives of ethnic, religious, and cultural minorities living in a “big ethnic culture”. These issues require the development of mechanisms of interethnic harmony in the society. In this context, the study aimed at investigating specific features of the individual’s ethnic identity determined by the degree of the ethnic diversity of their living environment is of relevance.

## 2. Materials and Methods

To define regions for the study, the comparative analysis of ethnic diversity of the Russian regions was conducted. The officially published statistical data were used in the analysis. The considered indicators included figures of the population by nationality in the constituent entities of the Russian Federation, i.e., a criterion of ethnic diversity was people belonging to ethnic groups. To quantify ethnic diversity, we used the Simpson diversity index applied to measure biodiversity. In the given study, it allowed us to assess a degree of possibility that two randomly selected representatives of the region will belong to two different ethnicities. Eighty-five regions of the Russian Federation were included in the analysis. A summary evaluation of every region was made through creating regional rankings according to Simpson’s index. It resulted in defining two regions for the study: the Sverdlovsk region (diversity index—0.263540) as a territory with average ethnic diversity and the Republic of Bashkortostan (diversity index—0.730376) as a highly diverse region in terms of ethnicity.

The study involved the sample from the Sverdlovsk region (N = 100) and the Republic of Bashkortostan (N = 100). The samples were of quota nature by gender, age, education, and the material wellbeing of the subjects. The following methods were applied in the study: the express questionnaire “Index of Tolerance” by G.U. Soldatova, O.A. Kravtsova, O.E. Khukhlaeva, L.A. Shaigerova (Ethnic Tolerance Scale), Z.V. Sikevich questionnaire allowing for identifying the intensity of ethnic “Self”, and the degree of ethnic status for the testees. The data were processed with the help of descriptive statistics methods, non-parametric Mann–Whitney U-test. The results were further analyzed via SPSS 20.0.

## 3. Results

The comparative analysis of the responses to Z.V. Sikevich’s questionnaire made it possible to reveal differences in the identification characteristics of the two groups compared. According to the results obtained, the respondents of the first group (the region with average ethnic diversity) equally exhibit global, citizen, and regional identity, while the respondents of the second group (the sample from the region with high ethnic identity) have predominantly citizen and ethnic types of self-identification (Table 1).

It should be noted that the respondents of Group 2 do not demonstrate a global type of identity (“I am a man of the world”) as well as affiliation with the European. The sample from the Republic of Bashkortostan involves not only the most numerous ethnic groups (Tatar, Bashkir) but also less represented ones (Mari, the Chuvash).

Also, differences in the intensity of ethnic self-identification in the compared groups were found. A bigger part of Group 2 feels an affiliation with a certain nationality with its own language, customs, and traditions, and belonging to several nationalities is perceived by 16.9% of the respondents. The subjects of Group 1 are characterized by a less stable ethnic identity, as a third of the group does not have it at all (Table 2).

It is noteworthy that the respondents from the region with high ethnic diversity exhibit no results when the subject does not feel an affiliation with any nationality. This fact allows for concluding that ethnic identity becomes significant only when two or more ethnic groups interact. This phenomenon is not relevant to ethnically homogeneous societies. One can treat ethnic identity as a component of acculturation in terms of how the person interacts with his ethnic group. It is necessary to separate ethnic identity from ethnicity. Ethnicity is a sociological concept associated with ethnic belonging in line with objectively established criteria (culture, language, nationality of the parents, place of birth). It is a society that ascribes ethnicity while the individuals experiences ethnic identity. It is them who shape it while constructing a social world based on ethnicity. However, ethnic identity is not limited to only this process. Identity, therefore, proves to be objective, and ethnicity is socially attributed.

Perhaps direct interactions with diverse ethnicities act as a “trigger” of the ethno-differentiating processes. The results below confirm this point (Table 3).

In other words, the respondents from the region with high ethnic diversity nationality is an essential characteristic of social categorization, meaning they never ignore it. For the respondents of Group 1, ethnicity is not significant and is diagnosed in the course of social cognition in situations with unpleasant subjects (i.e., it is actualized in attribute processes).

It is worth noting that the respondents from the region with high ethnic diversity are much more oriented to the preservation of their ethnic belonging, whereas 19% of the Sverdlovsk region sample expressed the desire to change their ethnic belonging if they had the chance.

Further analysis residing in the response comparison in line with the Ethnic Tolerance Scale of the questionnaire “Index of Tolerance” by G.U. Soldatova, O.A. Kravtsova, O.E. Khukhlaeva, L.A. Shaigerova served to clarify attitudes of the subjects to other ethnic groups (Table 4).

Therefore, the respondents from the region with high ethnic diversity are more selective and intolerant in their attitudes towards people of other ethnicities (general ethnic tolerance: M = 22.72, SD = 4.003) than the respondents from the region with average ethnic diversity (general ethnic tolerance: M = 27.12, SD = 5.143), Mann–Whitney U-test is 2513.500 where *p* = 0.000. This specific feature is accentuated in situations when a person of another ethnic origin is included in one’s inner circle of friends and family. These differences are statistically significant at the level of *p*< 0.05.

## 4. Discussion

The study conducted revealed several characteristics of ethnic self-awareness and ethnic identity in people from areas with different degrees of polyethnicity.

First, it has been revealed that the respondents living in a less ethno-diverse environment are characterized by global self-identity, the awareness of being a part of the world, and territorial identity, which is the awareness of being a country citizen or a resident of a certain city. The nature of identity of the control group is mostly ethnic.

Second, differences in the degree of sustainability and the intensity of ethnic self-identification of the subjects from regions with varying degrees of ethnic diversity have been found. The respondents from the polyethnic environment have clearly-defined ethnic belonging without the uncertainty of ethnic self-identification. It is likely the result of direct interactions with representatives of different cultures and ethnic groups, which provide the foundation for the respondents’ ethno-differentiation of themselves and others.

Third, essential differences in the significance of nationality for the compared groups have been indicated. For the subjects from the region with high ethnic diversity, the nationality of people around acts as an important attribute fixed in the course of interaction. The respondents from the region with average ethnic diversity pay attention to the nationality of other people in situations of unpleasant interaction. If a person is pleasant to deal with, then his nationality is ignored. Thus, for the respondents of the first group the nationality of other people is included in the process of social perception, while for the subjects of the second group, it is involved in the process of causal attribution.

Fourth, a higher ethnic tolerance was documented in the respondents from the region with moderate ethnic diversity.

The study allowed us to bring to light specific features of ethnic self-awareness in people whose living environments differ in the degree of ethnic diversity. In each case, any of essential features cannot predetermine the process of identification with any ethnic group. Such indicators as a language of communication and a native language, place of birth and place of living, and religion play a certain role in the formation of objective ethnic identity. However, at the same time, they do not pose it as an imperative. Objective ethnicity is a set of symbols and markers, which, in combination, create a particular sense of belonging to a community whose members can differ in various parameters and, at the same time, feel members of the same ethnic groups. Since signs of ethnos that can differentiate from others are subject to transformations, and it is not always possible to single out a collectivity as a totality, ethnic self-awareness remains practically a single criterion that can unite people in this group.

## 5. Conclusions

At present, the changes in the Russians’ mass consciousness, their consequences, and the transformation of ethnic identity present key problems of Russian society. Social interactions and conflicts in polyethnic environments can often result in rethinking the role of a person’s ethnicity and the transformation of their ethnic identity. One can argue that there is a social demand for studying the ethnic identity of modern Russia, and this knowledge will allow for tracing vectors of social development and corresponding changes.

The conducted study has not exhausted all the aspects of ethnic identity in the context of the impact of the environment. Prospects for further research entail the examination of the intensity of ethnic identity and ethnic self-awareness in respondents representing a prevailing ethnic group and ethnic minority in the regions.

## Figures and Tables

**Table 1:** The respondents’ identification self-sentiments (n1 = 100, n2 = 100), % of the subjects.

Group 1		Group 2	
Aspect of Identity	%	Aspect of Identity	%
Global citizen	17.08	Of the Russian Federation	22.06
European	6.13	Bashkir	15.23
Of the Russian Federation	24.71	Tatar	21.52
Yekaterinburg resident	36.72	Mari	22.44
Russian	14.36	Chuvash	18.45

**Table 2:** The individual ethnic self-identification of the respondents (n1 = 100, n2 = 100).

Aspect of Identity	Group 1	Group 2
Stable ethnic identity	41.73%	81.71%
Double self-identification	26.81%	18.29%
Lack of ethnic self-identification	31.46%	0

**Table 3:** Actualization of ethnic factor in the course of interaction (n1 = 100, n2 = 100).

Aspect of Identity	Group 1	Group 2
Ignore nationality of people around	44%	16%
Notice it if people are somehow unattractive	39%	18%
Notice it if people are somehow attractive	2%	19%
Notice nationality in any case	15%	47%

**Table 4:** The comparative analysis of the responses of the respondents from regions with varying ethnic diversity (n1 = 100, n2 = 100).

Statement	Group 1	Group 2	Mann–Whitney U-Test	Level of Significance
In mixed marriages, there are more problems than in marriages between people of the same nationality	M = 3.93; SD = 1.526	M = 3.24; SD = 1.658	3818.000	0.003
Attitudes to the Caucasian will improve if they change their behavior	M = 3.34; SD = 1.183	M = 3.3; SD = 1.115	4893.500	0.788
It is normal to think that your people are better than others	M = 4.42; SD = 1.334	M = 4.2; SD = 1.356	4458.500	0.172
I am ready to accept, as a family member, a person of any nationality	M = 3.59; SD = 1.393	M = 2.63; SD = 1.203	3023.500	0.000
I want to have friends of different ethnic origins	M = 4.11; SD = 1.476	M = 2.39; SD = 1.238	1972.500	0.000
It is hard to be good to some nations and peoples	M = 3.77; SD = 1.392	M = 3.53; SD = 1.425	4510.500	0.221
I can imagine a colored man as a close friend	M = 3.96; SD = 1.497	M = 3.42; SD = 1.505	3926.000	0.007

## References

[1] GIS and Agent-Based Modeling (2009). AAG Special Session: Modeling Geographic Complexity. https://www.gisagents.org/search?q=AAG+special+session%3A+Modeling+geographic+complexity.

[2] Evers H.-D., Genschick S., Schraven B. (2010). Constructing epistemic landscapes: Methods of GIS-based mapping. IUP J. Knowl. Manag..

[3] Evers H.-D., Gerke S., Menkhoff T. (2010). Knowledge clusters and knowledge hubs: Designing epistemic landscapes for development. J. Knowl. Manag..

[4] Holtug N., Mason A. (2010). Introduction: Immigration, diversity and social cohesion. Ethnicities.

[5] Shamsul A.B. From Conflict to Cohesion: The Paradigmatic Challenge in Analyzing Plural Societies in Southeast Asia, Malaysia as a Case Study. Proceedings of the International Conference on “Rethinking Realities, Reimagining Pluralism: Future Landscapes of Pluralism for Democratic Societies”.

[6] Luhmann N. (1984). Soziale Systeme. Grundriss Einer Allgemeinen Theorie.

[7] Menkhoff T., Evers H.-D., Chay Y.W. (2010). Governing and Managing Knowledge in Asia.

[8] Anderson B. (2006). Imagined Communities: Reflections on the Origin and Spread of Nationalism.

[9] Zotova O.Y., Tarasova L.V., Solodukhina O.S., Fairushina Z.F. Specific Features of Ethnic Identity in Children from Mixed Marriages. Proceedings of the International Conference on Psychology and Education (ICPE 2018).

[10] Chandler C.R., Tsai Y.M. (2001). Social factors influencing immigration attitudes: An analysis of data from the general social survey. Soc. Sci. J..

[11] Sears D.O., Henry P.J. (2003). The origins of symbolic racism. J. Pers. Soc. Psychol..

[12] Perelygina E.B., Zotova O.Y., Mostikov S.V. (2019). Gender Specificities of Ethnic Identity Transformation in Cross-National Interaction of the Russian. Proceedings of the 4th International Conference on Education Science and Development (ICESD).

[13] Berry J.W. (1999). Aboriginal cultural identity. Can. J. Nativ. Stud..

[14] Ajibade A., Hook J.N., Utsey S.O., Davis D.E., Van Tongeren D.R. (2016). Racial/ethnic identity, religious commitment, and wellbeing in African Americans. J. Black Psychol..

[15] Tajfel H., Turner J.C., Worchel S., Austin W.G. (1986). The social identity theory of intergroup behavior. Psychology of Intergroup Relations.

[16] Dockery A.M. (2010). Culture and wellbeing: The case of indigenous Australians. Soc. Indic. Res..

[17] Fleming J., Ledogar R.J. (2008). Resilience and indigenous spirituality: A literature review. Pimatisiwin.

[18] Houkamau C.A., Sibley C.G. (2011). Maori cultural efficacy and subjective wellbeing: A psychological model and research agenda. Soc. Indic. Res..

[19] Hughes M., Kiecolt J.K., Keith V.M., Demo D.H. (2015). Racial identity and well-being among African Americans. Soc. Psychol. Q..

[20] LaFromboise T.D., Hoyt D.R., Oliver L., Whitbeck L.B. (2006). Family, community, and school influences on resilience among American Indian adolescents in the upper Midwest. J. Community Psychol..

[21] Neblett E.W., Rivas-Drake D., Umaña-Taylor A.J. (2012). The promise of racial and ethnic protective factors in promoting ethnic minority youth development. Child Dev. Perspect..

[22] Brown D.J., Cober R.T., Keeping L.M., Levy P.E. (2006). Racial tolerance and reactions to diversity information in job advertisements. J. Appl. Soc. Psychol..

[23] Bisin A., Patacchini E., Verdier T., Zenou Y. (2011). Formation and Persistence of Oppositional Identities. Eur. Econ. Rev..

[24] Battu H., Zenou Y. (2010). Oppositional Identities and Employment for Ethnic Minorities: Evidence from England. Econ. J..

[25] Danzer A.M., Yaman F. (2013). Do Ethnic Enclaves Impede Immigrants’ Integration? Evidence from a Quasi-Experimental Social-Interaction Approach. Rev. Int. Econ..

